# Improving Gene-finding in *Chlamydomonas reinhardtii*:GreenGenie2

**DOI:** 10.1186/1471-2164-10-210

**Published:** 2009-05-07

**Authors:** Alan L Kwan, Linya Li, David C Kulp, Susan K Dutcher, Gary D Stormo

**Affiliations:** 1 Department of Computer Science and Engineering, Washington University in Saint Louis, Campus Box 1045, One Brookings Drive, Saint Louis, MO, USA; 2 Department of Genetics, Washington University School of Medicine, Campus Box 8232, 660 S. Euclid Avenue, Saint Louis, MO, USA; 3 Department of Computer Science, University of Massachusetts, 140 Governors Drive, Amherst, MA, USA

## Abstract

**Background:**

The availability of whole-genome sequences allows for the identification of the entire set of protein coding genes as well as their regulatory regions. This can be accomplished using multiple complementary methods that include ESTs, homology searches and *ab initio *gene predictions. Previously, the Genie gene-finding algorithm was trained on a small set of *Chlamydomonas *genes and shown to improve the accuracy of gene prediction in this species compared to other available programs. To improve *ab initio *gene finding in *Chlamydomonas*, we assemble a new training set consisting of over 2,300 cDNAs by assembling over 167,000 *Chlamydomonas *EST entries in GenBank using the EST assembly tool PASA.

**Results:**

The prediction accuracy of our cDNA-trained gene-finder, GreenGenie2, attains 83% sensitivity and 83% specificity for exons on short-sequence predictions. We predict about 12,000 genes in the version *v3 Chlamydomonas *genome assembly, most of which (78%) are either identical to or significantly overlap the published catalog of *Chlamydomonas *genes [1]. 22% of the published catalog is absent from the GreenGenie2 predictions; there is also a fraction (23%) of GreenGenie2 predictions that are absent from the published gene catalog. Randomly chosen gene models were tested by RT-PCR and most support the GreenGenie2 predictions.

**Conclusion:**

These data suggest that training with EST assemblies is highly effective and that GreenGenie2 is a valuable, complementary tool for predicting genes in *Chlamydomonas reinhardtii*.

## Background

A complete genome sequence facilitates the identification of all the genes in an organism and helps determine the set of functions encoded by those genes as well as the regulation of their expression. The identification of protein-coding genes can be approached both experimentally and computationally and the combination of approaches leads to the most complete catalog of genes [2]. Expressed sequence tags (ESTs) provide experimental evidence for the transcription of specific regions of the genome and significant similarity with known proteins in other organisms also provides evidence for the existence of a gene. However, both approaches have limitations that often preclude them from identifying the complete gene set. The exclusive use of the former would require a very large library of ESTs, obtained from a wide variety of environmental and developmental conditions, to ensure that all transcribed regions have been included. Identification based on homology will fail to identify genes that are novel to a particular species, or that are sufficiently diverged to make detection unreliable. *Ab initio *gene-finders provide a complementary gene identification method by predicting gene models based on the statistical characteristics of a representative set of protein-coding genes from the genome of interest.

Research using the unicellular green alga, *Chlamydomonas reinhardtii*, has provided important insights into many cellular processes that include flagellar assembly and motility, basal body assembly and positioning, phototaxis, gametogenesis and fertilization, circadian rhythms, photosynthesis, starch metabolism, and cell wall assembly [3-8]. *Chlamydomonas *is amenable to genetic analysis using classical techniques of tetrad analysis and complementation as well as molecular techniques of transformation and RNA interference [9].

The current catalog of genes for *Chlamydomonas reinhardtii *is based on a combination of experimental and computational approaches [1] where 44% of the 15,143 models in the catalog are derived from *ab initio *methods and the remainder use various evidence including similarity in other organisms and manual annotation. The inclusion of multiple *ab initio *gene-finders gives rise to complementary predictions by providing alternative models that can be used for experimental validation and may lead to the determination of true gene structures. Taken together, multiple methods may yield multiple correct predictions for genes with multiple alternate splice variants and a complementing gene-finder can also provide complete models for genes that are incomplete within an existing catalog and predict novel genes.

*Ab initio *gene-finders employ models that capture the essential features of gene structure that include sequence characteristics that distinguish exons and introns that include codon bias and feature length distributions as well as signal sequences that correspond to the splice sites that separate them [10,11]. Generalized hidden Markov models (gHMMs) are commonly used because gene structure can be represented in a probabilistic framework. Given a particular model of gene structure, the quality of predictions depends on the specific values assigned to the model parameters. Because these model parameters, such as codon bias and splice site patterns, vary between species, training a gene-finder on a representative set of example genes from the target species is closely related to the accuracy of the resulting predictions. The original GreenGenie [12] is a version of the Genie gene-finder [13] that was optimized for the prediction of genes in *Chlamydomonas*. The parameters for GreenGenie were obtained by training on only 71 genes with experimentally determined structure. GreenGenie provided more accurate predictions than other programs available at the time; it predicted 86 genes within 81 Kb and 443 Kb regions of *Chlamydomonas *genomic sequence and we extrapolated that number to predict between 12,215 and 16,414 genes in the *Chlamydomonas *genome. This prediction was recently corroborated [1]. GreenGenie facilitated gene identification in *Chlamydomonas *by many groups [14-16].

To improve the quality of gene prediction in *Chlamydomonas*, we used the EST assembly tool, Program to Assemble Spliced Alignments (PASA) [2], to assemble 167,613 *Chlamydomonas *EST sequences into protein coding gene models and trained the most recent version of the Genie *ab initio *gene-finder [13] on this larger set of *Chlamydomonas *gene models. The PASA pipeline begins by filtering and aligning input EST sequences onto a genome assembly. These ESTs alignments are then filtered further and clustered based on alignment compatibility. Finally, through a dynamic programming process, the EST alignment clusters are stitched into a set of consistent, non-overlapping EST assemblies [2]. PASA has been used for gene prediction in *Arabidopsis thaliana *[2], *Drosophila melanogaster *and *Homo sapiens *[17]. This larger training set improves the predictions made by the program, now called GreenGenie2, as determined on a set of 140 well-characterized *Chlamydomonas *genes that were not included in the training set and outperforms the most current published gene-finder trained for *Chlamydomonas*. Importantly, GreenGenie2 complements the existing *Chlamydomonas *gene catalog [1] by completing incomplete models and predicting new genes that were not previously identified.

## Results

### Constructing and evaluating a training-set of gene predictions from ESTs

PASA aligned 167,641 high-quality *Chlamydomonas *EST sequences onto the published genome assembly of *Chlamydomonas*, which is called *v3*, and assembled those alignments into 19,707 unique models. The set of PASA assembled models to be used for training were selected based on three criteria. First, the model must be complete; it must begin with an ATG codon and terminate with a stop codon (TAA, TAG or TGA). Second, the assembly must have a minimum open reading frame length of 270 bp. Third, the PASA model must lack similarity to the *gb140 *reference set of GenBank *Chlamydomonas *gene records (see Methods; see Additional file 1) and known transposable elements . These criteria reduce the 19,707 models to 2,384 models.

A similarity search of the 2,384 EST assembled models against the NCBI non-redundant database (NRdb) using NCBI BLAST (E < 1.0 × 10^-3^) was performed to assess the novelty of the assembled ESTs. 957 (40.1%) of the selected PASA assembled models align to an entry in NRdb (Table 1) and 482 (20.2%) of the remaining predictions have some overlap (see Methods) to models in the Frozen Gene Catalog [1], which we will refer to as *FGC07 *(see Methods). The remaining 945 (39.6%) complete PASA gene models in *v3 *are novel predictions identified by PASA EST assembly alone. We find that 835 of these novel models contain only a single exon. The quality of this large set of single-exon genes was evaluated by testing 13 randomly selected single exon models via RT-PCR. All 13 models yield product of the correct size with genomic DNA as the template and 10 of the 13 produce a fragment of the predicted size with cDNA as the template by RT-PCR (Table 2, Additional File 2). Given that the final set of 2,384 PASA assembled models are derived directly from 167,641 *Chlamydomonas *EST records and screened to have a complete compliment of gene features, this set of models is likely to provide an improved training set to optimize the parameters of the GreenGenie2 gene-finding program.

**Table 1:** Analysis of PASA gene models: Categorization of the 2384 PASA EST assembly gene models

**Class**		**N**
Alignment to NCBI NRdb		957/2384
Absent from the NCBI NRdb		1427/2384
Exact overlap in *FGC07*		222/1427
Partial overlap in *FGC07*		260/1427
No overlap in *FGC07*		945/1427
	Single exon	835
	Tested via RT-PCR	13
	Verified via RT-PCR	10

**Table 2:** Analysis of PASA gene models: RT-PCR testing of 13 novel, single exon PASA gene assemblies

Assembly ID	Outcome
3146_3724	Present in cDNA
5172_6168	Present in cDNA
8132_9749	Present in cDNA
9104_10933	Present in cDNA
9866_11843	Present in cDNA
11161_13363	Present in cDNA
11240_13451	Present in cDNA
11709_14017	Present in cDNA
14828_17825	Present in cDNA
16095_19351	Present in cDNA
14105_16951	Not present in cDNA
15620_18773	Not present in cDNA
14205_17074	Not present in cDNA

Present: A product of the correct size was found in samples by RT-PCR

Not present: No product was obtained by RT-PCR

Assembly ID numbers can be downloaded from . For primers used see Additional file 3

### GreenGenie2 is more accurate than GeneMark.hmm-ES 3.0

One primary purpose of gene-finders is to assist the user by accurately identifying genes in an isolated DNA segment that may be up to several kilobases in length. To evaluate the performance of GreenGenie2 on such short-sequence prediction queries we compared the performance statistics of GreenGenie2 and GeneMark.hmm-ES 3.0, the most recent, publicly available gene-finder trained specifically for *Chlamydomonas *[18].

Short-sequence prediction sensitivity and specificity of GreenGenie2 and GeneMark.hmm-ES 3.0 were computed for the total predictions made by each gene-finder using 140 genomic sequences obtained from the literature, referred to as *gb140 *(see Methods). At the whole-gene level, GreenGenie2 performs considerably better than GeneMark.hmm-ES 3.0. GreenGenie2 achieves sensitivity and specificity values of 0.51 (±0.10) and 0.47 (±0.11) while GeneMark.hmm-ES 3.0 sensitivity and specificity values are 0.31 (±0.10) and 0.24 (±0.09) (Table 3). A two-proportion *z*-test indicates that both differences are statistically significant (p < 0.001; see Methods). At the exon level, GreenGenie2 outperforms GeneMark.hmm-ES 3.0 with sensitivity and specificity values of 0.83 and 0.83 as compared to the corresponding values of 0.79 and 0.74 when using GeneMark.hmm-ES 3.0 (Table 3). The improvements in predictive accuracy made by GreenGenie2 are most obvious with initial and terminal exons (Table 3). At the nucleotide level, the least stringent assessment of prediction performance, GreenGenie2 shows an improvement of 2–3% over the GeneMark.hmm-ES 3.0 predictions (Table 3). These results indicate that GreenGenie2 is an improved *ab initio *gene-finder for *Chlamydomonas *and encouraged us to make whole-genome predictions on assembly *v3 *and compare them to the *FGC07 *catalog [1] with the goal of identifying new genes and improving the accuracy of the current gene models.

**Table 3:** Comparing GreenGenie2 and GeneMark.hmm-ES 3.0 in *gb140 *catalog

		GreenGenie2		GeneMark.hmm-ES 3.0	
		Sensitivity	Specificity	Sensitivity	Specificity

Gene Level	(n = 140)	0.51	0.47	0.31	0.24
Exon Level	(n = 1145)	0.83	0.83	0.79	0.74
Initial Exons	(n = 133)	0.65	0.60	0.50	0.40
Internal Exons	(n = 870)	0.87	0.88	0.84	0.84
Terminal Exons	(n = 133)	0.82	0.75	0.78	0.63
Single Exon	(n = 7)	0.71	0.62	0.00	0.00
Nucleotide Level	(n = 713682)	0.93	0.92	0.91	0.89

### GreenGenie2 models in v3 complement the Frozen Gene Catalog

GreenGenie2 predictions on *Chlamydomonas *genome assembly *v3 *were screened for a minimum coding length of 270 bp and against significant alignment to known transposable elements (see Methods). The final GreenGenie2 *v3 *catalog, *gg2v3*, consists of 12,387 predictions. The identical criteria applied to the *FGC07 *catalog leaves 12,320 predictions. All models were further classified as complete or incomplete based on the presence of start and stop codons (see Methods). All *gg2v3 *models are complete by construction. Of the 12,320 models in *FGC07*, only 67.7% are complete; the remaining 3,981 models lack a start codon, a stop codon or both.

Given the possible bias towards single-exon models in the GreenGenie2 training set, a comparison of single-exon models between *gg2v3 *and *FGC07 *was performed. In *FGC07*, 7.0% of complete models are single-exon genes and a similar proportion is observed in *gg2v3 *where 6.4% of the models are single-exon predictions. A two-proportion *z*-test (see Methods) indicates that there is no significant difference between the two proportions of single exon genes and that there is no bias towards the prediction of single-exon genes made by GreenGenie2.

**Figure 1:**
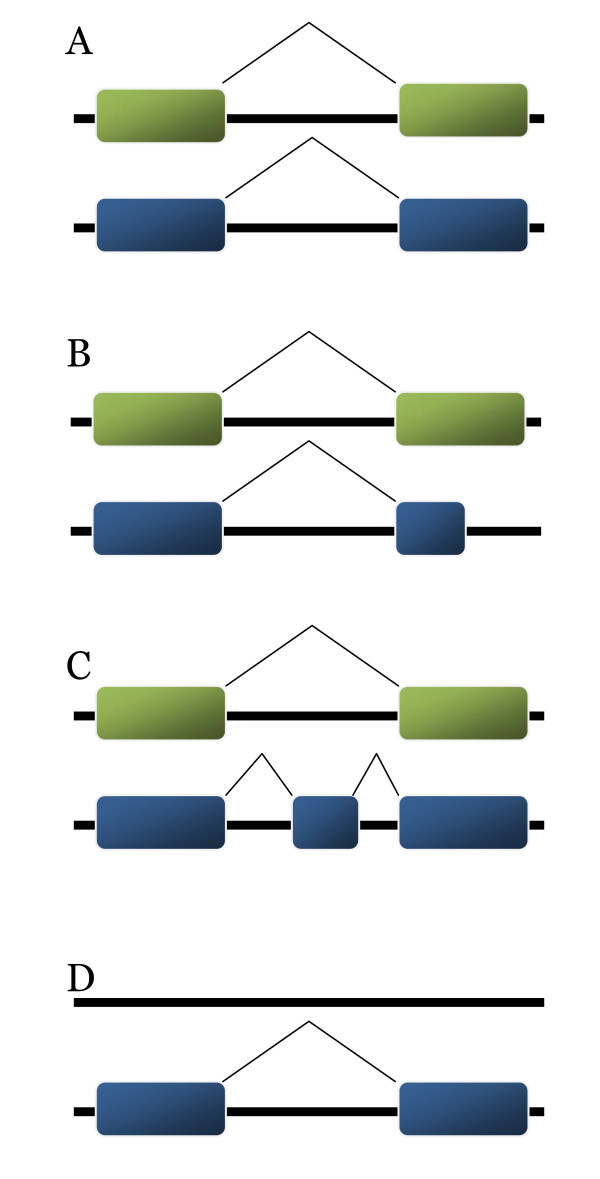
**Diagram of four classes of gene level interval overlaps**. Interval overlap analysis identifies four classes of predictions between the two catalogs. Grey tracks represent identical stretches of the genomic assembly. Either blue or green boxes distinguish exons in the two catalogs. (A) Predictions are exact overlaps; (B) Predictions show a partial gene overlap with an exact overlap of the 5' exon and partial overlap of the 3' exon; (C) Predictions show a partial gene overlap with an exact overlap of the terminal exons and an extra exon in one catalog but not the other; (D) A unique prediction present in one catalog but not present in the other catalog.

The *gg2v3 *gene catalog was compared to both the complete and incomplete partitions of *FGC07 *(Table 4) using interval overlap analysis. This analysis compares two lists of coding sequence coordinates that index a common underlying genome sequence and categorizes each prediction as consistent or conflicting (Figure 1; see Methods). Our analysis finds that 11% of the *FGC07 *models are predicted identically in *gg2v3 *and another 67% partially overlap with *gg2v3 *models (Table 4). The remaining 22% of *FGC07 *models have no overlap with *gg2v3 *models. Additionally, there are 2,859 (23%) *gg2v3 *models without interval overlaps to any model in *FGC07*.

**Table 4:** Comparison of *gg2v3 *and *FGC07 *catalog by overlap interval analysis

**Complete *FGC07 *models**		**Incomplete *FGC07 *models**	
Type of overlap	Count	Type of overlap	Count

Exact Overlap	1,324	Exact Overlap	0
Partial Overlap	5,425	Partial Overlap	2,826
No Overlap	1,574	No Overlap	1,149
Other	16	Other	16

Total	8,339	Total	3,981

Complete model: Any model that includes a starting ATG gene feature and terminates with a stop codon (TAA, TAG or TGA).

Incomplete model: Any model that lacks a start or stop codon or both.

Other: Models that interlaced overlaps and concatenated exact overlaps.

Predictions in *gg2v3 *that have partial interval overlaps to *FGC07 *models can be categorized into models with partially overlapping exons and models containing novel exons. Because Genie does not allow non-canonical splice sites, we determined the proportion of *FGC07 *exons that partially overlap *gg2v3 *exons with either canonical or non-canonical splice sites. Not all splice sites in *Chlamydomonas *follow the canonical rules [19]. However, allowing non-canonical splice sites might improve the sensitivity slightly, the marginal gain would come with the cost of many additional false positives.

15% of the partially overlapping *FGC07 *exons contain a non-canonical splice 5' site (GT) and 7% contain a non-canonical 3' splice site (AG). Therefore, about 20% of the non-identical, but overlapping exons between the *gg2v3 *and *FGC07 *catalogs are attributable to the fact that the GreenGenie2 model does not allow non-canonical splice sites. The set of partially overlapping models are of particular interest because they may include examples of alternative splicing as well as highlight incorrect models in each catalog. Each partially overlapping *gg2v3 *gene model with three or more exons (N = 6,885) was compared to the corresponding *FGC07 *model at the exon level. These exons were classified as initial, internal or terminal. The number of novel *gg2v3 *exons and partially overlapping exons was determined (see Additional file 3). The four largest groups have 1) partial overlaps for all three exon types (N = 761) and no new exons in the *gg2v3 *model, 2) an alternative initial exon (N = 480), 3) partially overlapping internal exons and both a novel initial and novel terminal exon (N = 461) and 4) an alternative terminal exon (N = 453). Overall, 28% of these models have new exon splice sites and no new exons in the *gg2v3 *model. Only 4% of the partially overlapping *gg2v3 *models have only novel exons (See Additional file 3). A small number of each of the partially overlapping models was tested using RT-PCR (see Methods). Figure 1B shows one type of model that has at least one exactly overlapping exon and at least one alternative exon terminus. No experimental support for any of the five *FGC07 *models tested was found, but support for four of the five corresponding *gg2v3 *models tested was found (Table 5, Additional File 4, Additinal File 5). Figure 1C illustrates the second type that has at least one exactly overlapping exon and at least one additional exon in the *gg2v3 *prediction that is absent from the *FGC07 *model. We find support for seven of the eight predictions tested (Table 5, Additional File 4, Additional File 5).

**Table 5:** Experimental analysis of 13 randomly selected predictions that differ between the *gg2v3 *and *FGC07 *catalogs

Models with alternate exon termini predicted in *gg2v3 *and *FGC07*			Novel exons predicted in *gg2v3 *not present in *FGC07*	
***gg2v3 *Gene ID**	**Support for *gg2v3***	**Support for *FGC07***	***gg2v3 *Gene ID**	***gg2v3 *support**

4t254	+	--	1t16	+
11t344	+	--	1t34	+
25t123	+	--	1t147	+
24t200	+	--	11t344	+
5t126	--	--	15t291	+
			30t106	+
			30t170	+
			3t257	--

+: A product of the correct size was found in samples by RT-PCR

--: No product was obtained by RT-PCR

*For primers see Additional files 4 and 5.

Predictions in one catalog that have no overlapping counterpart in the other catalog (Figure 1D) make up a significant proportion of both *gg2v3 *and *FGC07 *and may represent substantive sets of true genes that reflect the complementarity of the two catalogs. Our analysis finds that 22% (N = 2,723) of complete *FGC07 *models lack any overlap to models in *gg2v3 *and that 23% (N = 2,859) of *gg2v3 *models do not have interval overlap with any complete or incomplete model in *FGC07*. A small sample of predictions that are exclusive to each catalog was tested by RT-PCR. Four of the five *gg2v3 *predictions tested were supported by RT-PCR results (Table 6, Additional File 6, Additional File 7). Similarly, three of the five novel *FGC07 *predictions were supported by RT-PCR (Table 6, Additional File 6, Additional File 7). *In silico *analysis indicates that a majority of predictions exclusive to each catalog have EST or cross-species sequence similarity support or both. WU-BLASTP sequence similarity analysis indicates that 92.2% of gene models exclusive to *gg2v3 *align to some protein in the Eukaryotic Clusters of Orthologous Genes database (KOG) [20] or to some sequence in the *Chlamydomonas *EST database. Similarly, WU-BLASTP similarity analysis indicates that 94.5% of the *FGC07 *exclusive models are supported by evidence in the KOG or *Chlamydomonas *EST databases.

**Table 6:** Experimental analysis of 10 randomly selected predictions unique to the *gg2v3 *or *FGC07 *catalogs

Predictions exclusive to *gg2v3*		Predictions exclusive to *FGC07*	
*gg2v3 *Gene ID	Outcome	*FGC07 *Gene ID	Outcome

3t69	+	141597	+
19t170	+	181956	+
30t189	+	184911	+
76t11	+	141023	--
69t65	--	180935	--

+: A product of the correct size was found in samples by RT-PCR

--: No product was obtained by RT-PCR

*For primers see Additional files 6 and 7.

### GreenGenie2 is a robust, effective gene-finder across different genome assemblies

Our results in the previous section indicate that GreenGenie2 whole-genome predictions complement *FGC07 *[1] and suggest the potential value of including GreenGenie2 models in the annotation of future *Chlamydomonas *assemblies, so we used GreenGenie2 to predict a whole-genome catalog from the latest assembly of the *Chlamydomonas *genome, denoted as *gg2v4*. Sequence analysis of the two *Chlamydomonas *genome assemblies reveals that *v4 *contigs are seven times longer than *v3 *contigs on average, which highlights improved continuity in the *v4 *assembly compared to *v3 *assembly. GreenGenie2 predicts 11,135 models in the *v4 *assembly that satisfy the quality control constraints discussed previously. We mapped the *gg2v4 *models onto *v3 *scaffolds using BLAT [21] to facilitate the interval overlap analysis of the *gg2v4 *catalog with *gg2v3*. Only 20 of the *gg2v4 *models do not have matches in the *v3 *genome assembly. Conversely, 303 (2.4%) of the *gg2v3 *models do not have matches on the *v4 *assembly, which indicates a loss of some sequences in *v4 *compared to *v3*. 82.5% of the *gg2v4 *models (N = 9,184) map completely to a unique locus in *v3 *and likely represent loci that are shared between the *v3 *and *v4 *genome assemblies. 77% of these models are identical to models in *gg2v3 *despite the large changes in the genome contigs that are used for prediction. 21% of them have partial overlaps and only 1% is novel in the *gg2v4 *model set. Of the 17.1% of the *gg2v4 *models that do not map entirely to a single *v3 *locus, most of them (73%) have matches to two or more *v3 *loci, and the remainder contains additional sequences that do not occur on any *v3 *locus. The results indicate that the *gg2v4 *predictions from the updated *v4 *assembly are typically the same as the predictions on the shorter genome contigs of *v3*, which suggests that the predictions are not overly sensitive to the length of the contigs used as input. Furthermore, models that either were previously split across multiple contigs or were missing from the *v3 *assembly explain most of the differences. In both cases it appears that the updated *v4 *assembly has led to improved accuracy of the predicted gene catalog.

## Discussion

Determining genomic and EST sequence allows for the identification of the protein coding genes of a particular organism. We have used the information obtained from EST sequences to train the *ab initio *gene-finder Genie [13] on a filtered group of PASA assembled models that have both a start codon and a stop codon (complete) to create an accurate *ab initio *gene-finder for the GC-rich genome of the green alga *Chlamydomonas reinhardtii*.

The Program to Assemble Spliced Alignments (PASA) [2] was used to assemble *Chlamydomonas *EST sequences that were pre-aligned to the *v3 Chlamydomonas *genome assembly. This training set of 2,384 PASA assembled gene models has extensive biological evidence. Interval overlap analysis and homology search indicate that a majority of the PASA predictions align either to an existing *Chlamydomonas *gene model (21%) or have homologs in other organisms (40%). 39% of the PASA models are novel. Support for 10 of 13 novel predictions tested with RT-PCR suggests the potential for using the assembly of pre-aligned EST data as a primary basis of gene modeling, rather than as a supplementary source of predictive information.

One primary application of *ab initio *gene-finders is to accurately predict genes within short genomic sequences. Such short-sequence queries are often regions where the user has knowledge of a gene, but depends on the *ab initio *gene-finder to predict, confirm or correct the exon level structure of the gene. To test the short-sequence prediction accuracy of GreenGenie2, we compared the predictions of GreenGenie2 to the predictions of the most current, publicly available *ab initio *gene-finder trained for *Chlamydomonas*, GeneMark.hmm-ES 3.0 [18] on a set of 140 *Chlamydomonas *genomic sequences. Each of these genomic sequences contains a single known GenBank reference *Chlamydomonas *mRNA and the corresponding upstream (average length: 564 bp) and downstream (average length: 731 bp) flanking regions. Sensitivity and specificity of the two gene-finders was determined by comparing the prediction from each gene-finder against the reference GenBank annotation. Comparing the predictions on the gene level, GreenGenie2 is significantly more sensitive and specific (Table 3; p < 0.001) than GeneMark.hmm-ES 3.0. Results also indicate that GreenGenie2 outperforms GeneMark.hmm-ES 3.0 across all four types of exons (initial, internal, terminal and single), in particular, the initial and terminal exons.

Another application of *ab initio *gene-finders is the prediction of whole-genome gene catalogs. GreenGenie2 was used to predict a whole genome gene catalog on *Chlamydomonas *genome assembly *v3 *and this catalog, *gg2v3*, was compared to the existing *FGC07 *gene models by interval overlap analysis. The two catalogs predict a similar number of genes and a significant number of the models are identical. However, the two catalogs differ in several ways. First, there are a substantial proportion of complete *FGC07 *gene models that overlap but are not identical to *gg2v3 *models (54%). Exon level analysis of partially overlapping *gg2v3 *models shows that there are multiple causes (see Additional file 3). The four most frequent causes include partial exon overlap devoid of any new exons in *gg2v3*, models that are identical except in the initial exon, models where GreenGenie2 predicts entirely new initial and terminal exons and models that are identical except in the terminal exon. The third class reflects our observation that 32% of *FGC07 *models are incomplete. This analysis illustrates the range of complementarity that exists between the two catalogs. RT-PCR analysis found support for four out of five *gg2v3 *models (Figure 1B; Table 5), but failed to provide support for any of the five *FGC07 *models tested. In addition, seven of eight randomly selected *gg2v3 *models with additional exons that are absent from their *FGC07 *counterparts were validated by RT-PCR (Figure 1C; Table 5). Although the number of genes tested is small, the results suggest that GreenGenie2 complements the existing catalog by successfully identifying and correcting gene models that may be incorrect in the current *Chlamydomonas *annotation. Second, there is a set of *gg2v3 *predictions (N = 2,859) that is absent from *FGC07*, and a set of *FGC07 *predictions (N = 2,723) that is absent from *gg2v3*. We tested five randomly selected models from each set of exclusive predictions using RT-PCR and found support for four *gg2v3 *models and support for three of the *FGC07 *models tested. Furthermore, BLASTP alignment and EST alignment reveal that there is extensive support for almost all predictions that are absent from just *gg2v3 *(93.8%) or absent from just *FGC07 *(92.2%). These results indicate that each prediction method complements the other by identifying potentially true genes that are missing from the other catalog. Finally, GreenGenie2 completes 2,261 incomplete *FGC07 *models, which demonstrates another benefit of including GreenGenie2 whole-genome predictions into current and future *Chlamydomonas *gene catalogs.

The average contig length from assembly *v3 *to assembly *v4 *increases seven-fold, which indicates a greater degree of assembly continuity. The robustness of our gene-finder was tested across more continuous genome assemblies by using GreenGenie2 to predict a whole-genome gene catalog with the *v4 *genome assembly. If GreenGenie2 predictions were sensitive to the exact genome assembly used, and in particular if they varied substantially when the length of the genomic contigs changed, it would indicate unreliability in the predictions. However, we find that 77% of the *gg2v4 *models are identical to models in *gg2v3*, and most of the remainder overlaps significantly with the *gg2v3 *models. A large fraction of the differences are models where the *gg2v4 *predictions extend or merge models in *gg2v3 *based on the longer contiguous sequences in *v4*. These results are consistent with improvements in the updated assembly of *v4 *and with GreenGenie2 providing reliable predictions on a more contiguous genome assembly.

Overall, GreenGenie performance results on short-sequence and whole-genome predictions suggest that optimizing *ab initio *gene-finding parameters based on the assembly of a large collection of pre-aligned EST sequences as a rapid, low-cost and effective method by which *ab initio *gene-finders can be established.

## Conclusion

The *ab initio *gene-finder Genie was trained on a large set of complete PASA predicted gene models assembled from available *Chlamydomonas *EST sequence data. Short-sequence performance analysis indicates that GreenGenie2 is more accurate than the most recent *Chlamydomonas *gene-finder in the literature [18]. Interval overlap analysis between the GreenGenie2 *v3 *whole-genome catalog and the *FGC07 *catalog reveals that GreenGenie2 complements the current *Chlamydomonas *gene catalog [1] by accurately predicting new *v3 *gene models that are incomplete, incorrect or absent in *FGC07*. When GreenGenie2 was applied to the latest available *Chlamydomonas *genome assembly and the predicted *v4 *models were mapped back onto *v3 *scaffolds, GreenGenie2 appears to be robust against a seven-fold improvement in assembly continuity. These results illustrate a potential new application of EST sequence data to gene prediction and underscore the value of including the predictions of a fast, accurate *ab initio *gene-finder like GreenGenie2 into present and future catalogs. We have made the GreenGenie2 gene-finder described in this study available online. The submission form is available at .

## Methods

### Sequence datasets

This study uses the *Chlamydomonas *genome assembly version 3 . Genome assembly version 4 (*v4*) was obtained from Alan Kuo at the Joint Genome Institute.

Sequences longer than 1 Mb are pre-processed into shorter sequences prior to annotation by GreenGenie2. Pre-processing involves the removal of stretches of ambiguous nucleotides longer than 50 bp and treating the prefix and suffix as independent sequences. This pre-processing is advantageous for computational efficiency but to preserve maximal continuity in the assembly, all splitting events were chosen to minimize the final number of sequences. We found that requiring a minimum length of greater than 50 bp greatly increased the necessary number of splitting events. The *v3 *assembly was split from 1,557 sequences totaling 120,186,811 bases (~77.2 Kb/sequence) into 1,636 sequences totaling 120,076,271 bases (~73.4 Kb/sequence) following the removal of 110,540 ambiguous positions. The *v4 *assembly was split from 88 sequences totaling 112,305,447 bases (~1.3 Mb/sequence) into 218 sequences totaling 111,935,880 bases (~513.5 Kb/sequence) following the removal of 369,567 ambiguous positions.

A total of 140 experimentally verified *Chlamydomonas *annotations from GenBank  constitute a reference set for short sequence analysis and are referred to as *gb140 *(see Additional file 1). Initially, 222 GenBank records were retrieved by identifying records that indicated experimentally determined gene structure by direct sequencing of a complete cDNA and the genomic DNA and thus were not generated by automated assembly methods. The records were then filtered to remove genes with misannotated or missing start sites (N = 17), non-canonical splice sites (N = 46), misannotated or missing termination sites (N = 6) or open reading frames that are not multiples of three (N = 13). The included upstream and downstream flanking regions averaged 534 bp and 731 bp in length, respectively. The 167,613 EST records used to construct the PASA EST assemblies are from GenBank . All PASA EST assemblies were screened for significant alignment (BLAST E-value < 1.0 × 10^-20^) to *gb140 *before training to remove any bias in the subsequent short-sequence performance evaluation.

### Chlamydomonas gene catalogs

Three *Chlamydomonas *whole-genome catalogs were evaluated in this study: the GreenGenie2 whole-genome prediction on assembly *v3 *, the GreenGenie2 whole-genome prediction on assembly *v4 * and the Frozen Gene Catalog (*FGC07*) from Merchant *et al*. [1] (transcript file: ; model file: ). Prior to further analysis all models from all catalogs were screened for a minimum coding length of 270 bp and lack of significant alignment to known transposable elements . The choice of 270 bp as a minimum coding length is somewhat arbitrary, but there are very few verified genes shorter than this in *Chlamydomonas*. In *Saccharomyces cerevisiae*, recent studies show that there are about 200 genes (5%) that are less than 90 amino acids or 270 bp [22]. However in a genome that is 2/3 G+C like *Chlamydomonas*, prediction of genes 270 bp long or shorter will occur with a probability of 0.12. This probably in yeast about is about ten-fold lower (0.013). Thus, the inclusion of predicted genes that are less than 270 bp is likely to increase the number of falsely predicted genes greatly. Many models in *FGC07 *lack a start codon, a stop codon or both are thus considered incomplete models.

### Programs

Seven publicly available programs are used in this study. They are PASA [2], Genie [13], GeneMark.hmm-ES 3.0 [18], BLAT [21], WU-BLAST [23], NCBI-BLAST  and Primer3 [24]. EST sequence assembly was performed using PASA (Program to Assemble Spliced Alignments). The initial EST alignments were performed by PASA using the built-in GMAP algorithm option [25]. The GreenGenie2 program is based on the latest version of the Genie gene-finder [13]. Genie implements a general hidden Markov model (gHMM) to predict protein-coding regions in genomic DNA. The most recently published gHMM gene-finder trained specifically for *Chlamydomonas *is GeneMark.hmm-ES 3.0 [18], which is used in this study as the short-sequence performance benchmark for GreenGenie2. Unless otherwise stated, all sequence alignments were performed using WU-BLAST and significant alignments are those with BLAST E-value < 1.0 × 10^-5^. PASA EST assembly alignment to the NCBI non-redundant database (NRdb) was conducted by NCBI using NCBI-BLAST (default BLAST E-value < 1.0 × 10^-3^). Alignment of *v4 *models onto *v3 *was performed using BLAT with the -fine and -maxIntron = 5000 program options invoked. All primers used in this study were designed using Primer3 [24].

### Short-sequence prediction performance evaluation

The evaluation of predictions requires independent and high quality annotated test sequences against which predictions are compared to determine sensitivity and specificity statistics and a quantitative evaluation of prediction accuracy. When comparing the predicted genes for a given test sequence to the reference annotation of that sequence, the predicted structure can be evaluated at three different levels: nucleotide accuracy, exonic accuracy and whole gene accuracy [26]. Whole gene accuracy is the most stringent level because a prediction is correct only when the prediction matches the reference at every position; a single mismatched exon boundary is an error and renders the entire prediction incorrect. Nucleotide accuracy is the least stringent level; each individual nucleotide is either correctly or incorrectly labeled as coding or non-coding. At each level, predictions are classified as either true positive, false positive, true negative or false negative. True positives and true negatives are those regions where the predicted structure agrees with the reference annotation in coding and non-coding regions respectively. Conversely, false positives and false negatives are those regions where the predicted structure does not agree with reference annotations in non-coding and coding regions respectively. Sensitivity is defined as the ratio of true positives to actual positives. Greater sensitivity on the gene level indicates that the prediction method being evaluated misses fewer genes. Specificity is defined as the proportion of all predictions that are true positives. Greater specificity at the gene level indicates that there are fewer wrong predictions being made by the prediction method under evaluation. By determining the different relative ratios of each of the four categories above, it is possible to gauge the inherent accuracy of a set of predictions and to compare the predictive performance across different sets of gene predictions. Short-sequence prediction performance of GreenGenie2 is performed by submitting the genomic sequences corresponding to each of the 140 reference annotations in *gb140 *to both GreenGenie2 and GeneMark.hmm-ES 3.0. Each sequence yields a single set of predictions from each of the gene-predictors. Standard averaged sensitivity and specificity ratios are computed on the nucleotide, exon and gene levels by the Tally.pl and BaseCounts.pl, utilities that are included as a part of the Genie software package . Statistical significance of differences between two ratios is computed by a two-proportion *z*-test that compares the corresponding ratios for a given confidence level from each of the two independent predictions. All such comparisons in this study are computed using a confidence level of 0.99.

### Interval overlap analysis

Whole-genome predictions are compared using interval overlap analysis of predicted models and evaluated for accuracy and complementarity. The interval overlap analysis of gene features is performed by directly comparing two lists of coding sequence coordinates indexed on a common genome assembly. Coding nucleotides are classified as either overlapping or not overlapping. A coding nucleotide is overlapping if and only if that position is annotated as coding in both predicted models, otherwise the nucleotide is not overlapping. Exons are classified into three classes: exact, partial and novel. An exon for which every nucleotide is aligned is classified as an exact overlap. An exon that is not classified as an exact overlap but has at least thirty consecutive bases that overlap is classified as a partial overlap. An exon that is neither exact nor partial is classified as extra in the original catalog and absent in the other catalog. A gene is classified into three classes: exact, partial and novel. A gene for which every exon is classified as exact is classified as exact. A gene for which every exon is classified as novel is classified as novel. All other genes are classified as partial, which indicates that the two predictions overlap but are not identical. Differing predictions between two catalogs can then be targeted for subsequent testing via RT-PCR and other *in silico *validation methods.

### PCR and RT-PCR

A small subset of novel predictions with non-exact overlaps was tested by RT-PCR. Two classes of predictions were tested: predictions that overlap but are not exact and predictions that are exclusive to each catalog. To verify exons whose intron boundaries do not agree between two catalogs, one primer aligns to the overhanging region of each of the two partially aligned exons and the other primer aligns to a nearby exon that is exactly overlapping between the two catalogs. RT-PCR with these primers unambiguously indicates which prediction (if either) is correct, or whether both predicted genes are correct and arise from alternative splicing. The designed primers were also used in genomic DNA PCR to verify that they amplify the correct regions of interest. For genomic DNA PCR, crude *Chlamydomonas *DNA was prepared. A toothpick-tip-full of *Chlamydomonas *cells was lyszed in 10 μL lysis buffer (10 mM Tris-HCl, pH 8.8, 50 mM KCl, 2 mM MgCl_2_, 0.1% Triton-100, 1 mg/mL proteinase K) at 58°C for 1 hr followed by 95°C 30 min to denature the proteinase K. Cell debris was collected by a 10 sec centrifugation and 0.5 μL of the supernatant were used in a 10 μL PCR reaction. Total RNA from wild-type vegetative *Chlamydomonas *cells was prepared as previously described [27]. Total RNA (30 μg) was treated with 2 units of RNase-free DNase I (New England Biolabs, Ipswitch, MA) to remove contaminating genomic DNA from the sample. One μg of total RNA was used for cDNA synthesis with or without the addition of SuperScript II reverse transcriptase (Invitrogen, Carlsbad, CA) in a 20 μl reaction. The same reaction mix without reverse transcriptase serves as the control for the presence of genomic DNA contamination. 0.5 μL of cDNA synthesis products was used in a 10 μl PCR reaction with RedTaq DNA polymerase (Sigma, St. Louis, MO) according to the manufacturer's protocol. PCR conditions used were the following: 95°C 2 min, followed by 30 cycles of 95°C 15 sec, 53°C 15 sec, and 72°C 1 min, and ending at 72°C for 2 min.

## Authors' contributions

ALK and GDS conceived of this study. ALK participated in its design, carried out the computational studies, performed the overall analysis and drafted the manuscript. LL carried out the PCR and RT-PCR experiments. DK participated in the training of the gene-finder, SKD and GDS participated in the design of the study and its coordination and helped to draft the manuscript. All authors read and approved the final manuscript.

## Supplementary Material

Additional file 1 **Tabulated List of NCBI Protein ID codes for *gb140***. A table listing the NCBI Protein ID codes that comprise the set *gb140*. Click here for file

Additional file 2 **List of Primers: 13 Novel PASA Assemblies.** A table of primers used to test 13 randomly chosen, single-exon PASA EST assembled gene models. Click here for file

Additional file 3 **Histogram of exon overlap gg2v3 models with partial overlaps in FGC07.** A histogram detailing the different classes of exons that are overlapping between gg2v3 and FGC07 gene models. Click here for file

Additional file 4 **List of Primers: *gg2v3 *Predictions with partially overlapping exons**. A table of primers used to test five pairs of partially overlapping gene models between *gg2v3 *and *FGC07 *with alternative exon termini. Click here for file

Additional file 5 **List of Primers: *gg2v3 *Predictions with novel exons**. A table of primers used to test eight *gg2v3 *gene models with extra exons when compared to *FGC07*. Click here for file

Additional file 6 **List of Primers: *gg2v3 *Exclusive Genes**. A table of primers used to test five *gg2v3 *gene models that have no overlapping model in *FGC07*. Click here for file

Additional file 7 **List of Primers: *FGC07 *Exclusive Genes**. A table of primers used to test five *FGC07 *gene models that have no overlapping model in *gg2v3*. Click here for file
