# Supplementary material for: Improving Gene-finding in Chlamydomonas reinhardtii:GreenGenie2
Source: BMC Genomics. 2009 May 7;10:210. doi: 10.1186/1471-2164-10-210 (PMC2694837; doi:10.1186/1471-2164-10-210)
Supplement: Additional file 1 — Tabulated List of NCBI Protein ID codes for gb140. A table listing the NCBI Protein ID codes that comprise the set gb140. [file 1471-2164-10-210-S1.doc]

**Additional file 1 – Tabulated List of NCBI Protein ID codes for *gb140***

| AAB23258.2 | AAM01186.2 | CAA37638.1 | AAD28474.1 |
| --- | --- | --- | --- |
| AAK77552.1 | AAR04931.1 | AAC49416.1 | AAK06774.1 |
| AAF43040.1 | CAD60538.1 | AAG30934.1 | AAM15777.1 |
| AAK32150.1 | AAQ12259.1 | AAK68064.1 | AAO45104.1 |
| AAR20884.1 | AAT37069.1 | AAK70872.1 | AAO48940.1 |
| AAY86155.1 | AAC03784.1 | AAK70874.1 | AAQ16277.2 |
| CAA48233.1 | AAG45420.1 | AAK84866.1 | ABC02019.1 |
| AAA82610.1 | AAO25117.1 | AAL31495.1 | AAG29840.1 |
| AAB71841.1 | AAK14648.1 | AAN77901.2 | AAT40991.1 |
| CAE17329.1 | AAM19664.1 | AAS07042.1 | AAN87017.1 |
| AAB71840.1 | AAQ95705.1 | AAS89977.1 | AAQ19847.1 |
| AAK01720.1 | AAA57316.2 | CAA65356.1 | AAA84971.1 |
| AAK82666.1 | AAB39840.1 | CAC19676.1 | AAP30010.1 |
| AAR23425.1 | AAC49887.1 | ABC49916.1 | AAR82947.1 |
| AAF65221.1 | AAC49888.1 | AAP12520.1 | CAB56598.1 |
| AAL75576.1 | AAD45352.1 | AAP12521.1 | AAK38270.1 |
| AAC08533.1 | CAD24295.1 | AAM44041.1 | AAK54060.1 |
| AAC08534.1 | AAO86687.1 | AAG45421.1 | AAF34540.1 |
| AAD39433.1 | AAD27871.1 | AAK37411.1 | AAG33634.1 |
| AAG40000.1 | AAF17595.1 | AAM88388.1 | AAM23012.1 |
| AAP21826.1 | AAF73174.1 | AAY56335.1 |  |
| AAQ83687.1 | AAM15771.1 | CAA41039.1 |  |
| AAD10324.1 | AAR82949.1 | AAW67003.1 |  |
| AAM88387.2 | AAD27849.1 | ABG38184.1 |  |
| CAF25319.1 | AAL28128.1 | AAM18057.1 |  |
| AAK32117.1 | ABK56835.1 | AAQ16626.1 |  |
| AAL35726.1 | CAA44066.1 | AAD55941.1 |  |
| AAL79816.1 | AAB60274.1 | ABK34486.1 |  |
| AAF36402.1 | AAO53242.1 | AAG37909.1 |  |
| ABB88568.1 | AAP57169.1_v1 | AAP83163.1 |  |
| AAB95196.1 | AAP57169.1_v2 | CAD32174.1 |  |
| AAM23259.1 | AAY56333.1 | AAT38474.1 |  |
| AAM23262.1_v1 | AAY56334.1 | AAT38475.1 |  |
| AAM23262.1_v2 | AAL37900.1 | AAB00730.2 |  |
| AAM44130.1_v1 | AAP85534.1 | AAD52203.1 |  |
| AAM44130.1_v2 | CAE46409.1 | AAC37438.2 |  |
| AAQ55462.1 | AAO61143.1 | AAD38856.1 |  |
| AAS07044.1 | AAL73208.1 | AAM43910.1 |  |
| AAG33633.1 | AAN01224.1 | AAD50464.1 |  |
| AAK77219.1 | AAC27525.1 | AAK14341.1 |  |
