# Supplementary material for: Improving Gene-finding in Chlamydomonas reinhardtii:GreenGenie2
Source: BMC Genomics. 2009 May 7;10:210. doi: 10.1186/1471-2164-10-210 (PMC2694837; doi:10.1186/1471-2164-10-210)
Supplement: Additional file 2 — List of Primers: 13 Novel PASA Assemblies. A table of primers used to test 13 randomly chosen, single-exon PASA EST assembled gene models. [file 1471-2164-10-210-S2.doc]

Additional file 2 –List of Primers: 13 Novel PASA Assemblies

| Assembly ID | Left Primer | Right Primer | Predicted Length |
| --- | --- | --- | --- |
| 3146_3724 | GCC GCA ACA CTG TTT GTG TA | AAA GCA TGT GTC CCC TCG T | 138 |
| 5172_6168 | TGC ACT AAG TCC GAA CAC GA | CCA TGT AGG CGG GAG AGT AA | 143 |
| 8132_9749 | AGA GCA AGC GAG TTC GAG AG | GTG AGC AAA GGC ACT TAG GC | 136 |
| 9104_10933 | GCC GAA ATT CCA AGT CAA GA | TGC CTG GTG TAA TCG TGG TA | 168 |
| 9866_11843 | CCA AGT GCC ACT CCA TAG C | ATC GTG GAC TGA GCG GTG T | 130 |
| 11161_13363 | CCC ACA AAC ACA TGA GAA TCC | TCC AGT GCA GTT CCA TCT GA | 169 |
| 11240_13451 | CGG AGT GAC CAA TAG GGT TC | CAC CTC GAG GCT TAG CTG TC | 149 |
| 11709_14017 | ACC ACA CCT TTT TGC GGT AA | GAT GCA GTG TGG CAG AGG TA | 139 |
| 14828_17825 | GTC TGG TAG CTT CCG AGC AG | ACC CCC TCA GGA ACG TGT AT | 139 |
| 16095_19351 | TAC TAC GAT GCG GAT GTG GA | GGA TTT GGT TCA GGG AGG AG | 150 |
| 14105_16951* | AGA CAT GAA CGT CCC CTC AC | CAG CGC AAC TCT GAC AGA CA | 158 |
| 15620_18773* | GGT TGT ATA CGC TGC TGC TG | GGC AAA GCC TAC ACA GCT TC | 150 |
| 14205_17074* | TCT TCT CGT TTA GCG CGT TT | CGC ACG CTA TAC GTC TCT CC | 147 |

*failed to yield predicted product
