# Supplementary material for: Improving Gene-finding in Chlamydomonas reinhardtii:GreenGenie2
Source: BMC Genomics. 2009 May 7;10:210. doi: 10.1186/1471-2164-10-210 (PMC2694837; doi:10.1186/1471-2164-10-210)
Supplement: Additional file 3 — Histogram of exon overlap gg2v3 models with partial overlaps in FGC07. A histogram detailing the different classes of exons that are overlapping between gg2v3 and FGC07 gene models. [file 1471-2164-10-210-S3.doc]

**Additional file 3 – Histogram of exon overlap *gg2v3* models with partial overlaps in *FGC07***

Exon level interval overlap analysis identifies three types of exons in *gg2v3* models with partial overlaps in *FGC07*: initial, internal and terminal. Each of the three exon types are represented in a three digit code. The rightmost digit corresponds to the terminal exon, the middle position corresponds to all internal exons and the leftmost digit corresponds to the initial exon. Each digit is assigned a value of 0, 1, 2 or 3. A value of 0 at a given position indicates that all exons of that type are exact for every gene in that category. A value of 1 indicates that there is one or more occurrence of partial exon overlap of exons in the position’s exon type and no novel exons predicted in *gg2v3*. A value of 2 indicates that there is one or more occurrences of a whole new exon predicted in *gg2v3* that is absent in the overlapping *FGC07* model in the exon type corresponding to that position for all genes with that code and no partially overlapping exons between the two catalogs. A value of 3 indicates that there is one or more occurrences of both partially overlapping exons and extra exons in *gg2v3* when compared to the model in *FGC07*. (E.g. *gg2v3* models in the class 111 have one or more partially overlapping exons in *FGC07* of all three exon types and no occurrences of extra exons predicted; *gg2v3* models in the class 100 have exact exon matches across all exons in the model except for the initial exon).
