# Supplementary material for: Improving Gene-finding in Chlamydomonas reinhardtii:GreenGenie2
Source: BMC Genomics. 2009 May 7;10:210. doi: 10.1186/1471-2164-10-210 (PMC2694837; doi:10.1186/1471-2164-10-210)
Supplement: Additional file 4 — List of Primers: gg2v3 Predictions with partially overlapping exons. A table of primers used to test five pairs of partially overlapping gene models between gg2v3 and FGC07 with alternative exon termini. [file 1471-2164-10-210-S4.doc]

**Additional file 4 –List of Primers: *gg2v3* Predictions with partially overlapping exons**

| Gene ID | Left Primer | Right Primer | Predicted Length |
| --- | --- | --- | --- |
| 4t254 | ACA ACG GCA CCA TCA TCA AT | GCC GGT TAC GGT GAT GTT | 123 |
| 4t254_143087† | CTA CAA CGG CAC CAT CAT CA | AGC CAG CGT GCC GTA CTC | 103 |
| 11t344 | GTT CTG CTG CCT CTG GTC AT | GTC CCA CTC GAC CCT CCT | 100 |
| 11t344_169877† | GTT CTG CTG CCT CTG GTC AT | TTG ATT GCG TCA ATG GAA AC | 105 |
| 25t123 | GTG TCC ATC TGC CTG CAC | TTC AGC GGG CAC ACA TTT AC | 90 |
| 25t123_104389† | GTG TCC ATC TGC CTG CAC | TGT GCA CTT GCA ATG GAG TAT | 106 |
| 24t200 | AGA TGA TTG TGT TCC GAC AGG | GGC GTC GCT TAC GTC CAG | 104 |
| 24t200_195571† | CCC CTC CTA CCA GAT GAT TG | GTT TGG GTG AAA GCG GAC T | 100 |
| 5t126* | ATC TCT TCA CGG CAC CTT C | TGT GTG CAG GTA AGG GTG AG | 148 |
| 5t126_186782†* | ATG TCT TCA CGG CAC CTT C | GGG GAT GGC TGT CAT GTA CT | 143 |

*failed to yield predicted product

†*gg2v3* gene id and corresponding protein ID in *FGC07*
