# Supplementary material for: Improving Gene-finding in Chlamydomonas reinhardtii:GreenGenie2
Source: BMC Genomics. 2009 May 7;10:210. doi: 10.1186/1471-2164-10-210 (PMC2694837; doi:10.1186/1471-2164-10-210)
Supplement: Additional file 5 — List of Primers: gg2v3 Predictions with novel exons. A table of primers used to test eight gg2v3 gene models with extra exons when compared to FGC07. [file 1471-2164-10-210-S5.doc]

**Additional file 5 –List of Primers: *gg2v3* Predictions with novel exons**

| Gene ID | Left Primer | Right Primer | Predicted Length |
| --- | --- | --- | --- |
| 1t16 | GCG TAT CGC CCA AAT GAA | GCG GTG ATG ATG TGT TTG TC | 100 |
| 1t34 | ACG AGG ACG ACT ACG ACG AC | GTC CTT GAG AAG GCG GAA C | 102 |
| 1t147 | CTG GTG TCC GTG TAC ATT GC | TCG GGT GCC ATC CAG TAG | 198 |
| 11t344 | ACC GAC TGC GAA GAC TGT G | CCT TGC TCT GCA GCA ACC | 107 |
| 15t291 | CCT GAC GCC TAC GAC AAG TT | GGA ACA CGG ACT CCA GAG C | 128 |
| 30t106 | ACA ACC AGT CGC AGA AGG AG | CTG TCC ACA GCT CTG ACG TG | 181 |
| 30t170 | CAT TGG AGA CCA GGA CGA G | GTC TCG CGT GTG AGT GTT TG | 106 |
| 3t257* | GTC ACC GCG GAC CTA CTG | GAC TCT CAG CAG CTT CTC TCG | 140 |

*failed to yield predicted product
