# Supplementary material for: Improving Gene-finding in Chlamydomonas reinhardtii:GreenGenie2
Source: BMC Genomics. 2009 May 7;10:210. doi: 10.1186/1471-2164-10-210 (PMC2694837; doi:10.1186/1471-2164-10-210)
Supplement: Additional file 6 — List of Primers: gg2v3 Exclusive Genes. A table of primers used to test five gg2v3 gene models that have no overlapping model in FGC07. [file 1471-2164-10-210-S6.doc]

**Additional file 6 –List of Primers: *gg2v3*** Exclusive Genes

| Gene ID | Left Primer | Right Primer | Predicted Length |
| --- | --- | --- | --- |
| 3t69 | CAG CTC CAC CAA CAA CGA G | ATC ACC ACC AGC TTG CTG TC | 115 |
| 19t170 | GCT GGT GCT GGT GTT AAA TG | GTG TCC GCT AGC CGC TTA AT | 136 |
| 30t189 | ATC AGC CTG GAG GAG CTG | TGA CAC CGT GGA TCT TAC ACA | 119 |
| 76t11 | CCT GGG CTG GGA CTT TTC | GTC CTG GTA GCG CTC ACA TC | 110 |
| 69t65* | AAC TCC GGG AGC TTT ACA CA | TTT GGA CCA AGA CCT GAA GC | 108 |

*failed to yield predicted product
