# Supplementary material for: Improving Gene-finding in Chlamydomonas reinhardtii:GreenGenie2
Source: BMC Genomics. 2009 May 7;10:210. doi: 10.1186/1471-2164-10-210 (PMC2694837; doi:10.1186/1471-2164-10-210)
Supplement: Additional file 7 — List of Primers: FGC07 Exclusive Genes. A table of primers used to test five FGC07 gene models that have no overlapping model in gg2v3. [file 1471-2164-10-210-S7.doc]

**Additional file 7 –List of Primers: *FGC07*** Exclusive Genes

| Gene ID | Left Primer | Right Primer | Predicted Length |
| --- | --- | --- | --- |
| 141597 | GTG CAA CTC GGC CTG GAT | GTG GGC GAG AAT GTG GTT AG | 103 |
| 181956 | CCT GAA CTG CAT CAT CCA CA | ATC ATG ACC TCA CGC GTC TC | 152 |
| 184911 | GCG CAG GCA TTA CAG GTC | GGA GCC TCC TGG TGA TGA G | 112 |
| 141023* | GTG GAT CCC GAG GCT GTC | ATG CCG ACA TCG TGA ACT G | 104 |
| 180935* | GTG CTG TCC AGG CAA AGG | TGC TAG CAG CTC TGA CAC CT | 168 |

*failed to yield predicted product
